# Thermoelectric Properties of Zn-Doped YbMg_1.85−*x*_Zn*_x_*Bi_1.98_

**DOI:** 10.3390/ma17050973

**Published:** 2024-02-20

**Authors:** Simin Wei, Nailing Qin, Guiying Wu, Zhengbing Xu, Lei Miao, Xiyong Chen, Jialin Yan

**Affiliations:** 1 School of Resources, Environment and Materials, Guangxi University, Nanning 530004, China; wsm@st.gxu.edu.cn (S.W.); 18377613069@163.com (N.Q.); wuguiyingw5@163.com (G.W.); xuzhb@gxu.edu.cn (Z.X.); xiyongchen@gxu.edu.cn (X.C.); 2 State Key Laboratory of Featured Metal Materials and Life-Cycle Safety for Composite Structures, Nanning 530004, China; miaolei@gxu.edu.cn; 3 Guangxi Key Laboratory for Relativity Astrophysics, School of Physical Science and Technology, Guangxi University, Nanning 530004, China

**Keywords:** YbMg_2_Bi_2_, Zintl compounds, microstructure, the bipolar effect, thermoelectric properties

## Abstract

Bi-based YbMg_2_Bi_1.98_ Zintl compounds represent promising thermoelectric materials. Precise composition and appropriate doping are of great importance for this complex semiconductor. Here, the influence of Zn substitution for Mg on the microstructure and thermoelectric properties of *p*-type YbMg_1.85−*x*_Zn*_x_*Bi_1.98_ (*x* = 0, 0.05, 0.08, 0.13, 0.23) was investigated. Polycrystalline samples were prepared using induction melting and densified with spark plasma sintering. X-ray diffraction confirmed that the major phase of the samples possesses the trigonal CaAl_2_Si_2_-type crystal structure, and SEM/EDS indicated the presence of minor secondary phases. The electrical conductivity increases and the lattice thermal conductivity decreases with more Zn doping in YbMg_1.85−*x*_Zn*_x_*Bi_1.98_, whereas the Seebeck coefficient has a large reduction. The band gap decreases with increasing Zn concentration and leads to bipolar conduction, resulting in an increase in the thermal conductivity at higher temperatures. Figure of merit *ZT* values of 0.51 and 0.49 were found for the samples with *x* = 0 and 0.05 at 773 K, respectively. The maximum amount of Zn doping is suggested to be less than *x* = 0.1.

## 1. Introduction

Exploring sustainable energy and improving low energy conversion efficiency are top priorities to solve the increasing energy shortage. As core materials for power generation devices in thermoelectric (TE) technology, TE materials allow the interconversion of waste thermal energy and electrical energy without additional energy consumption and have been intensively investigated because of their numerous applications in industrial waste heat recovery, power generation, solid state cooling, and space probes [1,2]. The performance of a TE material is generally evaluated by the term “figure of merit *ZT*”, which is given as *ZT* = *S*^2^*σT/κ*, where *S*, *σ*, *κ*, and *T* represent the Seebeck coefficient, the electrical conductivity, the thermal conductivity, and the absolute temperature, respectively. *κ* generally comes from two sources, i.e., the electronic contribution *κ_e_*, which is proportional to *σ* based on the Wiedemann–Franz relationship, and the lattice contribution *κ_l_* [3]. High-performance TE materials are expected to possess low *κ,* and one of the effective solutions for high *ZT* is to search for alloy materials with intrinsic low *κ* values. On the other hand, the balance between *σ* and *S* is vital since these parameters are coupled and normally work in opposite directions. The electronic transport properties can be better described by the term “power factor *PF*”, defined as *S*^2^*σ*, and high *PF* is required [4]. Zintl compounds have complex crystal structures that often lead to intrinsic low lattice thermal conductivity, and their covalent bonding within the polyanionic framework allows maintenance of reasonable carrier mobility. These characteristics of Zintl compounds make them fit well into the concept of phonon–glass electron–crystal [5,6]. High *ZT* can be achieved by balancing the conflicting electron and phonon transports [7,8,9,10].

Among the Zintl phases, the typical 1-2-2 type Sb-based AB_2_Sb_2_ compounds (where A stands for Yb, Eu, Ca, Sr, and Ba; B stands for Cd, Zn, and Mg) have demonstrated promising TE performance in the 500–800 K temperature range [8,11]. Gascoin et al. reported a maximum *ZT* of 0.56 at 773 K for Ca*_x_*Yb_1−*x*_Zn_2_Sb_2_ with *x* = 1 [8]. Later, Wang et al. obtained a peak *ZT* of 1.2 at 700 K for YbCd_2−*x*_Zn*_x_*Sb_2_ with *x* = 0.4 [11]. With a layered CaAl_2_Si_2_ structure, the 1-2-2 compounds consist of a layered covalently bonded polyanionic framework that enables high mobility for charge transport (“electron–crystal”) and weakly bonded cations that facilitate phonon scattering (“phonon–glass”) [5,6,12,13,14]. The *ZT* values of the pristine 1-2-2 phases are generally lower than 0.5, and enhanced TE performance with maximal *ZT* values above unity can be finely tuned and achieved by doping or alloying for the formation of solid solutions. Strategies for enhancing *ZT* include orbital engineering [15,16], band engineering [17,18,19], as well as point defect or microstructure engineering [20,21]. The analogous Bi-based 1-2-2 type Zintl phases have received less attention compared to Sb-based compounds. May et al. studied the TE properties of CaMg_2_Bi_2_, EuMg_2_Bi_2_, and YbMg_2_Bi_2_ between 2 and 650 K with the polycrystalline samples prepared by melting and hot pressing and found that YbMg_2_Bi_2_ exhibited the highest *ZT* value approaching 0.4 near 625 K [22]. Recently, Shuai et al. demonstrated that for samples synthesized using high-energy ball milling and hot pressing, a significantly enhanced *ZT* of approximately 1.3 can be achieved at 873 K for (Eu_0.5_Yb_0.5_)_1−*x*_Ca*_x_*Mg_2_Bi_2_ samples with *x* = 0.4 [18]. The authors suggest that the nanostructures as well as the alloying effect contribute to the high *ZT.* Shuai et al. found through composition optimization that YbMg_2_Bi_1.98_ with a minor Bi deficiency exhibits improved TE performance [23]. Subsequent studies have focused either on substitution at the cationic A^2+^ site of AMg_2_Bi_2_ Zintl compounds [16,17] or substitution at either site in the anionic ${\left[{Mg}_{2}{Bi}_{2}\right]}^{2-}$ framework [24,25]. It has been shown that the equivalent substitution of Zn for Cd or Mg has successfully led to a greater *ZT* in YbCd_2−*x*_Zn*_x_*Sb_2_ [11], CaMg_2−*x*_Zn*_x_*Bi_1.98_ [25], and YbMg_2−*x*_Zn*_x_*Sb_2_ [26] owing to the enhanced phonon scattering induced by point defects and/or orbital alignment. Co-doping of Zn at the Mg site and Li, Na, K [25], or Yb [27] at the Ca site in CaMg_2_Bi_1.98_ samples synthesized using ball milling and spark plasma sintering (SPS) were explored by Guo et al. It is reported that a competitive peak *ZT* of approximately 1.0 was achieved for Ca_0.995_Li_0.005_Mg_1.9_Zn_0.1_Bi_1.98_ at 873 K [25] and Ca_0.65_Yb_0.35_Mg_1.9_Zn_0.1_Bi_1.98_ at 773 K [27]. Guo et al. further studied the defect formation energy, band structure, phonon dispersion, and TE properties of CaMg_2_Bi_2_ with Zn doping at either the Ca or the Mg site experimentally and theoretically using density functional theory [16]. They revealed that Zn doping at the Ca site is more effective in increasing the carrier concentration and thus *σ* due to more remarkable lowering of the formation energy of Ca vacancy than that of Mg vacancy [16]. Based on the literature, we synthesized samples of YbMg_2−*x*_Zn*_x_*Bi_1.98_ with an actual composition of approximately YbMg_1.85−*x*_Zn*_x_*Bi_1.98_ using high-frequency induction melting followed by ball milling and SPS. The doping of Zn at the Mg site increases *σ* and lowers *κ_l_*, and these effects are explained by the electronegativity and mass differences between Zn and Mg. However, the bipolar effect occurs at lower temperatures with increasing Zn concentrations, leading to a lower *ZT*. The overall effects of Zn partially replacing Mg alone on the *PF* and *ZT* are less satisfactory. The maximum amount of Zn doping is suggested to be less than *x* = 0.1. 

## 2. Materials and Methods

Polycrystalline samples with a nominal composition of YbMg_2−*x*_Zn*_x_*Bi_1.98_ (*x* = 0, 0.2, 0.3, 0.4, 0.6) were prepared from high-purity granules of Yb (99.95%) from Xingtai Zhongyan, Beijing, China, and Mg (99.95%), Bi (99.99%), and Zn (99.995%) from Zhongnuo New Materials, Beijing, China. The starting elements were kept and weighed in an argon-filled glovebox where the levels of O_2_ and moisture were kept below 0.01 ppm. Then, the elements were placed in a corundum crucible covered with a lid. The reactants in the corundum crucibles were melted in an argon-filled quartz tube using high-frequency induction melting (Shanghai Renjun High Frequency Equipment, Shanghai, China). The melting process was controlled by adjusting the heating electric current and the heating time. The heating process started with a low current for a few minutes and then shifted to higher current holding for a few more minutes for thorough melting. In this work, the appropriate electric current and holding time were identified and adopted after several attempts to obtain single-phase samples. To provide homogeneous powder for sintering, the as-melted samples were powdered in an agate mortar and moved to a stainless-steel jar (with milling balls) filled with argon inside the glovebox. Ball millings of the samples were then carried out for 3 h in a planetary ball mill (QM-3SP4, Nanjing, China). In an argon-filled glovebox, the resulting powders were taken out of the stainless-steel jar and placed into a graphite die with carbon paper underneath to allow for easy demolding. The densifications of the samples with a diameter of 15 mm were then conducted at 873 K for 5 min using SPS (LABOX-225, SINTER LAND INC., Niigata-ken, Japan) in vacuum at an axial pressure of 50 MPa.

Powder X-ray diffraction (XRD) patterns were collected from 10° to 110° 2*θ* in increments of 0.02° in a step-scan mode with Cu *Kα* radiation (Rigaku D/Max 2500 V, Tokyo, Japan) for structure characterization. The XRD data were refined for quantitative phase analysis with the Rietveld method using the Fullprof program [28]. Field-emission scanning electron microscope (FE-SEM) (Hitachi SU8020, Tokyo, Japan) and energy-dispersive X-ray spectroscopy (EDS) (Oxford X-MAX80, Oxford, UK) were carried out to examine the microstructures and compositions of the SPS sintered samples. The microstructures were observed on polished and freshly fractured surfaces, separately. The chemical compositions of the samples and the phases were the average of at least three EDS point measurements. The samples were found to be Mg and Zn deficient. The actual sample compositions were measured to be YbMg_1.85_Bi_2.05_ (denoted as ~YbMg_1.85−*x*_Zn*_x_*Bi_1.98_ with *x* = 0 thereafter), YbMg_1.75_Zn_0.05_Bi_1.94_ (*x* = 0.05), YbMg_1.77_Zn_0.08_Bi_1.93_ (*x* = 0.08), YbMg_1.69_Zn_0.13_Bi_1.94_ (*x* = 0.13), and YbMg_1.63_Zn_0.23_Bi_1.87_ (*x* = 0.23) due to the evaporation of Mg and Zn during the melting process. Rectangular specimens for electronic transport measurements and disc specimens for thermal measurements were cut from the SPS sintered disks. Simultaneously measurements of *σ* and *S* were performed using an Advance Riko ZEM-3M10 (Yokohama, Japan) from room temperature up to 773 K under high-purity helium. The thermal diffusivity *D* measurements were conducted in a NETZSCH LFA 467 HyperFlash (Selb, Germany) under a nitrogen atmosphere employing a laser flash method. Experimental measurements of the specific heat capacity *C_P_* of partial samples were carried out using PtRh crucibles with Al_2_O_3_ lining under an argon atmosphere up to 803 K in a NETZSCH DSC 404C (Selb, Germany) according to the sapphire method. The measured *C_P_* values were compared with the calculated temperature-dependent *C_P_* values determined using the corrected Dulong–Petit law from Agne et al. [29] for each measured composition. The bulk densities *d* of the SPS sintered samples were measured using a METTLER TOLEDO AG285 (Shanghai, China) based on Archimedes’ principle. The thermal conductivity *κ* was then determined using the equation *κ = DC_p_d*.

## 3. Results and Discussion

### 3.1. Phase Analysis

**Figure 1:**
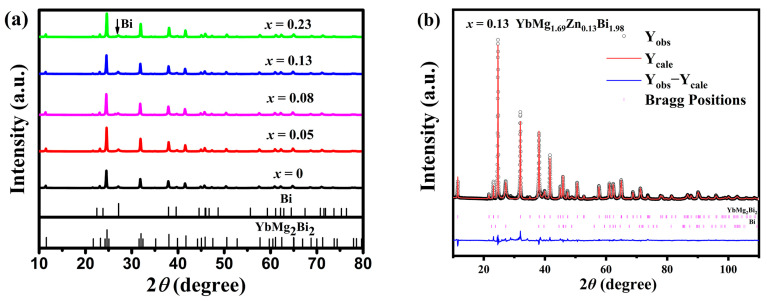
(**a**) Powder XRD patterns of the YbMg_1.85−*x*_Zn_*x*_Bi_1.98_ (*x* = 0, 0.05, 0.08, 0.13, and 0.23) samples and (**b**) Rietveld refinement of the XRD pattern for the sample with *x* = 0.13.

The room temperature XRD patterns of YbMg_1.85−*x*_Zn*_x_*Bi_1.98_ samples with *x* = 0, 0.05, 0.08, 0.13, and 0.23 are presented in Figure 1a. The majority of the diffraction peaks for all the samples indicate the YbMg_2_Bi_2_ phase, which is trigonal CaAl_2_Si_2_-type structure with space group $P\bar{3}m1$ (No. 164) [30,31]. A diffraction peak at ca. 27.1° 2θ is observed in all samples, as indicated by the arrow in Figure 1a, and is identified as the strongest peak of the Bi phase. Additional weak peaks at ca. 28.7 and 34.2° 2θ with a peak height less than two for samples with *x* ≥ 0.08 are detected, indicating the existence of other trace phases. Structure refinements for the 1-2-2 and Bi phases were carried out on the XRD patterns of the samples. For the 1-2-2 phase with a CaAl_2_Si_2_-type structure, the Mg/Zn atoms co-occupy the 2*d* site (1/3, 2/3, *z*), and their site occupancies (Occ. Mg/Zn) were fixed according to the EDS measured compositions. The occupancies of Mg and Zn on the 2*d* site are less than 1 for the Mg/Zn-deficient samples. Figure 1b shows the Rietveld refinement of the XRD pattern for the sample with *x* = 0.13. The structural parameters for the 1-2-2 phase, the amounts (*m* in wt. %) and the X-ray theoretical densities (*ρ*) for both the 1-2-2 and Bi phases derived from Rietveld refinement, the bulk densities (*d*) measured using Archimedes’ method, and the relative theoretical density (% T. D.) are listed in Table 1. It is seen that with more Zn doping, the unit cell parameters *a* and *c* and the volume *V* decrease slightly. This is reasonable in consideration of a covalent radii of 1.379 Å for Zn and 1.598 Å for Mg according to Ref. [32]. The amounts of the secondary phases obtained from the refinements are found to be around 10–17 wt. % of the samples, as further revealed by SEM and EDS.

**Figure 2:**
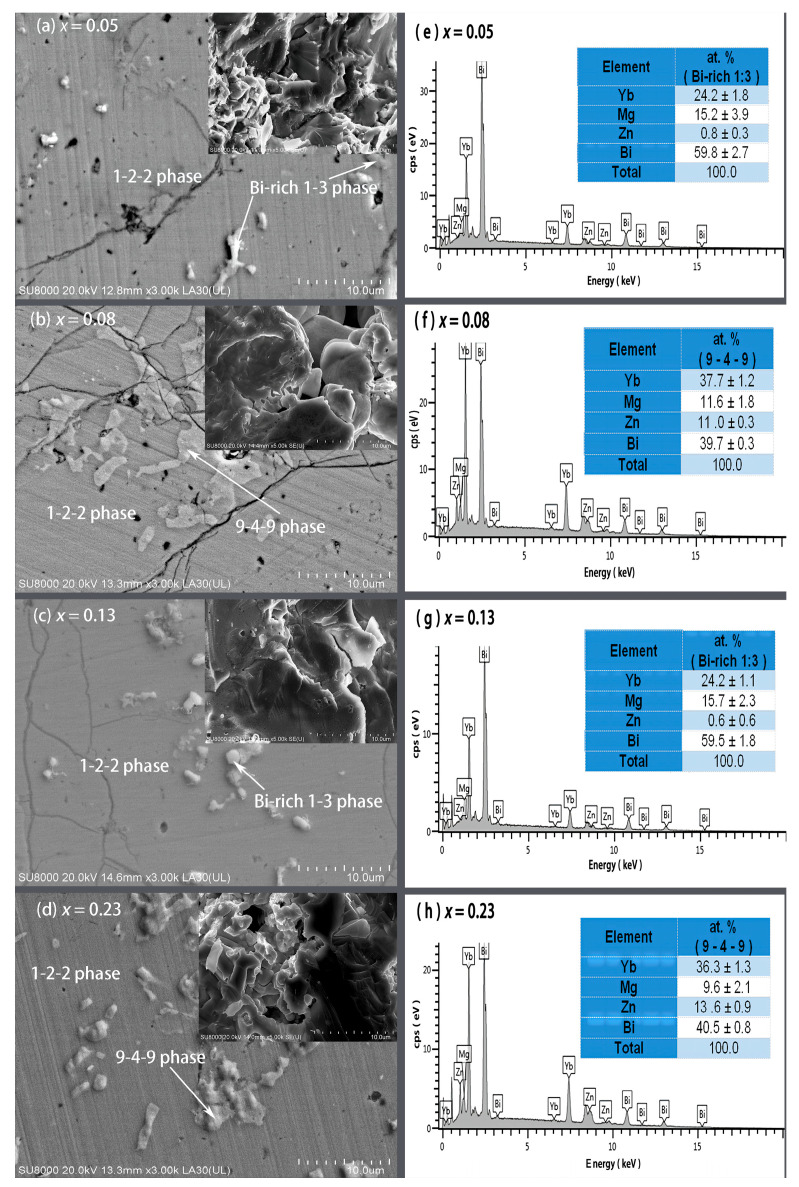
FE-SEM images on the polished and fractured (inset) surfaces for YbMg_1.85−*x*_Zn_*x*_Bi_1.98_ with (**a**) *x* = 0.05, (**b**) *x* = 0.08, (**c**) *x* = 0.13, and (**d**) *x* = 0.23 and the corresponding EDS spectra of the secondary phases in YbMg_1.85−*x*_Zn_*x*_Bi_1.98_ with (**e**) *x* = 0.05, (**f**) *x* = 0.08, (**g**) *x* = 0.13, and (**h**) *x* = 0.23.

Figure 2 presents the FE-SEM images on polished and fractured surfaces and EDS spectra with average compositions of the secondary phases for the YbMg_1.85−*x*_Zn*_x_*Bi_1.98_ samples with *x* = 0.05, 0.08, 0.13, and 0.23. Based on the EDS compositional analysis on the polished surfaces, the matrix for all the samples are the 1-2-2 phase, and the secondary phases are revealed to be in the form of the Yb_9_(Mg, Zn)_4.5−δ_Bi_9_ phase (denoted as 9-4-9 phase) and Bi-rich phase (denoted as Bi-rich 1–3 phase) with a likely formula of Yb(Mg, Zn)_0.5−0.7_Bi_2.3_ (Figure 2e–h). The fractured morphology for the sample with *x* = 0.05 exhibits a lamellar dense structure, whereas pores and voids were observed in samples with *x* ≥ 0.08, which may be caused by the evaporation of Mg and Zn. The bulk densities (*d*) were measured to be 7.130, 7.197, 7.271, 7.314, and 7.159 g cm^−3^ for *x* = 0, 0.05, 0.08, 0.13, and 0.23, respectively. From the X-ray theoretical densities of the 1-2-2 phase *ρ*^122^ and the Bi phase *ρ^Bi^*, the theoretical densities of the samples were estimated using the relation $\rho =\rho^{122}\rho^{Bi}/\left(\rho^{122}m^{Bi}+m^{122}\rho^{Bi}\right)$, and the % T. D. was then obtained (Table 1). It is noted that the values of % T. D. exceed 98% except for the sample with *x* = 0. The sample with *x* = 0.05 has the highest % T. D., which is in good agreement with the observed fractured morphology.

### 3.2. Electronic and Thermal Transport Properties

**Figure 3:**
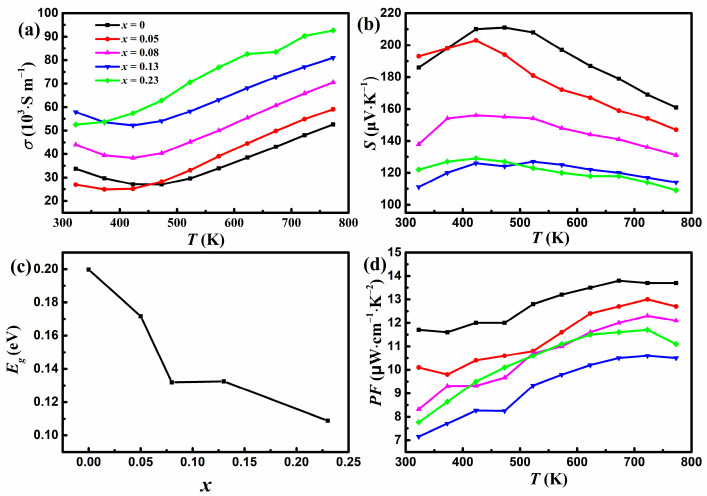
Variations in (**a**) the electrical conductivity and (**b**) the Seebeck coefficient with temperature. (**c**) Variation in *E_g_* with Zn concentration *x*. (**d**) Variation in the power factor with temperature for YbMg_1.85−*x*_Zn_*x*_Bi_1.98_ (*x* = 0, 0.05, 0.08, 0.13, 0.23).

Figure 3a shows the variation of *σ* with temperature for YbMg_1.85−*x*_Zn*_x_*Bi_1.98_ (*x* = 0, 0.05, 0.08, 0.13, 0.23). The *σ* values for samples with *x* ≤ 0.13 exhibit an initial decrease until 423 K and then an upward trend as the temperature increases, whereas *σ* values for samples with *x* = 0.23 increase monotonously with temperature. Similar behaviors have been reported for Ca_1−*x*_Yb*_x_*Mg_2_Bi_2_ [23] and CaMg_2−*x*_Zn*_x_*Bi_1.98_ [25]. The initial decrease in *σ* indicates the typical behavior of degenerate semiconductors, and the increase in *σ* above 423 K exhibits the characteristics of intrinsic semiconductors. From the intrinsic region above 523 K, the band gap *E_g_* can be estimated using Arrhenius law $\sigma \left(T\right)=exp\left(-E_{g}/2k_{B}T\right)$, where *k_B_* is Boltzmann’s constant. Values of 0.16, 0.16, 0.125, 0.09, and 0.07 eV were obtained for *x* = 0, 0.05, 0.08, 0.13, and 0.23, respectively. The *E_g_* value of 0.16 eV for *x* = 0 is close to that of 0.18 eV for YbMg_2_Bi_2_ reported in Ref. [18]. The σ increases as more Zn replaces Mg, which can be understood by the electronegativity of the elements in the ${\left[{Mg}_{2-x}{Zn}_{x}{Bi}_{1.98}\right]}^{2-}$ polyanionic framework. The electronegativities of Mg, Zn, and Bi are 1.31, 1.65, and 2.02, respectively. The introduction of Zn with greater electronegativity strengthens the covalent bond nature through the formation of Zn-Bi bonding in the polyanionic framework and thus enables good mobility of charge carriers, resulting in better electron transport [6].

Figure 3b shows that all the samples have positive values of *S*, indicating a *p*-type conductive behavior. The variations in *σ* and *S* for *x* = 0 are consistent with the results of YbMg_2_Bi_2_ in Ref. [23]. In accordance with *σ*, temperature-dependent *S* initially increases, exhibiting a degenerate semiconducting behavior, and then decreases with increasing temperature, showing a bipolar effect due to the intrinsic carrier excitation. The value of the maximum Seebeck coefficient *S*_max_ for *x* = 0 is 211 μV K^−1^ and drops to 129 μV K^−1^ for *x* = 0.23. The *S* vs. T curve with a peak *S*_max_ can be used to estimate the band gap *E_g_* with the Goldsmid–Sharp formula *E_g_* = 2*e|S|*_max_*T*_max_, where *T*_max_ is the temperature corresponding to *S*_max_ and *e* is the electron charge [33]. A relationship between *E_g_* and the concentration of doped Zn is shown in Figure 3c. It is found that although the *E_g_* values calculated from the *S* vs. T curves are greater than those obtained from the *σ* vs. T curves, they show similar trends. *E_g_* decreases with increasing Zn concentrations. The narrowing of *E_g_* is in favor of the increase in charge carrier concentration and is therefore beneficial for *σ*. Meanwhile, narrow band gap semiconductors are prone to intrinsic carrier excitation. The narrowing of *E_g_*, which coincides with the variations in *σ* and *S* with Zn concentrations, reveals that the occurrence temperature of the bipolar effect decreases with increasing Zn concentrations. The overall *S* under bipolar conduction can be expressed using the following formula [34]:

(1) $$S=\frac{{S_{e}\sigma }_{e}+{S_{h}\sigma }_{h}}{\sigma_{e}+\sigma_{h}}$$

where subscripts *e* and *h* express electron and hole carriers, respectively. The decline in *S* with Zn concentration can be attributed to the competition between the positive contribution *S_h_* from holes and the negative contribution *S_e_* from the electrons. As shown in Figure 3d, all the Zn-doped samples exhibit lower *PF* values than the undoped samples. The doping of Zn for Mg results in a decrease in *PF* up to *x* = 0.13 because the remarkable reduction of *S* predominates in the calculation of *PF*. The obtained maximum *PF* of 13.8 μW cm^−1^ K^−2^ for *x* = 0 at 673 K is close to the value reported for YbMg_2_Bi_2_ at 700 K [23].

**Figure 4:**
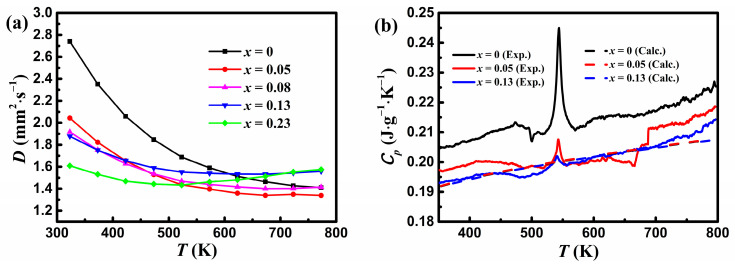
Variations in (**a**) the thermal diffusivity for YbMg_1.85−*x*_Zn_*x*_Bi_1.98_ (*x* = 0, 0.05, 0.08, 0.13, 0.23) and (**b**) the measured and calculated specific heat capacity with temperature. The dashed lines represent the calculated *C_p_* obtained from the corrected Dulong–Petit law in Agne et al. [29].

Figure 4a shows the variation in the thermal diffusivity *D* with temperature for YbMg_2−*x*_Zn*_x_*Bi_1.98_. It is seen that *D* for samples with *x* ≤ 0.08 decreases monotonously with increasing temperature, whereas *D* for samples with *x* ≥ 0.13 increases at higher temperatures, implying a bipolar diffusivity due to the decrease in the band gap. *D* for *x* = 0.23 shows the lowest value at 323 K but the highest value at 773 K. This phenomenon has been observed in Ref. [23]. It has been reported that a widening band gap and/or increasing carrier concentration by doping Eu or Yb at the Sm^2+^ site in SmMg_2_Bi_2_ [17], trace monovalent ion Ag^+^ at the Mg^2+^/Zn^2+^ sites in YbMg_2−*x*_Zn*_x_*Sb_2_ [26], or Sn at the Bi site in Yb_0.8−*x*_Ca*_x_*Mg_0.2_Mg_2_Bi_1.96_ [35] can lead to an increase in the temperature for the intrinsic carrier excitation, thus effectively resulting in suppressed bipolar diffusion.

Figure 4b shows variations in the measured specific heat capacity with temperature for samples with *x* = 0, 0.05, and 0.13. A peak at ca. 544 K that corresponds to the melting point of Bi is assigned to the Bi phase, which is similar to that reported for YbMg_2_Bi_2_-based alloys in Ref. [23]. The experimental *C_p_* decreases with Zn concentration as expected from the estimated values based on the Dulong–Petit limit. A Debye temperature of 309 K and a *C_p_* value of 121 J mol^−1^ K^−1^ (i.e., 0.189 J g^−1^ K^−1^) at 300 K for YbMg_2_Bi_2_ were obtained from the measurements of *C_p_* over the temperature range of 1.8–300 K [36]. As a comparison, the calculated *C_P_* values obtained using the corrected Dulong–Petit law from Agne et al. [29] for measured EDS compositions are also presented in Figure 4b. It is evident that the calculated *C_P_* is in good agreement with the experimental *C_p_*. In view of the negligible effect of the minor variation of *C_p_* values on the total *κ*, the calculated *C_P_* values are used in this paper to determine the total *κ* using the relationship *κ = DC_p_d*. 

**Figure 5:**
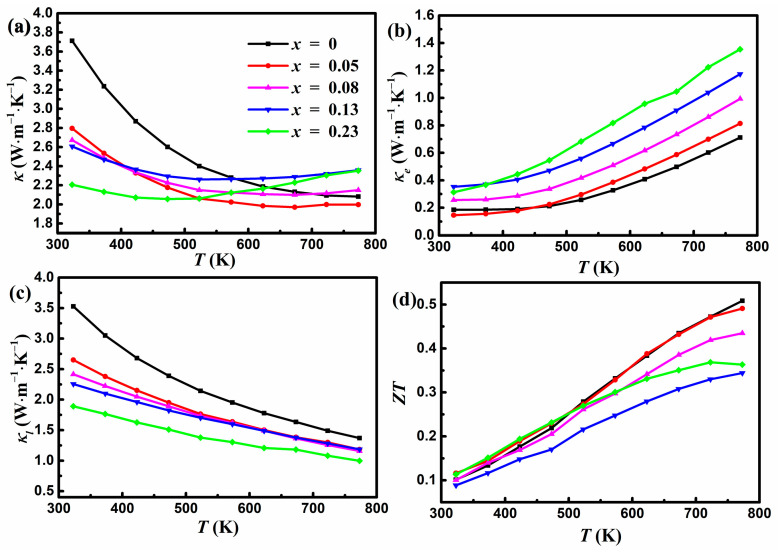
Variations in (**a**) the total thermal conductivity, (**b**) the electronic thermal conductivity, (**c**) the lattice thermal conductivity, and (**d**) *ZT* with temperature for YbMg_1.85−*x*_Zn_*x*_Bi_1.98_ (*x* = 0, 0.05, 0.08, 0.13, 0.23).

As shown in Figure 5a, the total *κ* exhibits a similar temperature-dependent tendency as *D*. *κ* at 323 K is reduced from 3.7 W m^−1^ K^−1^ for *x* = 0 to 2.2 W m^−1^ K^−1^ for *x* = 0.23, a reduction of about 41%. However, *κ* at 773 K increases slightly from 2.1 W m^−1^ K^−1^ for *x* = 0 to 2.4 W m^−1^ K^−1^ for *x* = 0.23, with a minimum *κ* of 2.0 W m^−1^ K^−1^ for *x* = 0.05. It is noted that the total *κ* of the undoped YbMg_1.85_Bi_2.05_ sample is slightly greater compared to those given in Ref. [23], where samples were prepared using high-energy ball milling and then hot pressing or SPS. This can be ascribed to the existence of coarse secondary phases in this work and no thorough ball milling to form the nanostructures. It is known that TE properties vary sensitively with different preparation methods [5]. Mechanical alloying effectively enhances boundary scattering and increases the densities of point defects and nanostructures, resulting in a reduced *κ*. Figure 5b presents the *κ_e_* calculated using *κ_e_ = LσT*. The Lorentz number *L* can be estimated using the following equation [37]:

(2) $$L=1.5+exp\left[-\frac{\left|s\right|}{116}\right]$$

In Equation (2), the unit of *L* is 10^−8^ W Ω K^−2^, and that of *S* is μV K^−1^. It is seen that *κ_e_* increases with the increase in Zn concentration, which is consistent with the change in *σ*. The corresponding *κ_L_* presented in Figure 5c was obtained by subtracting *κ_e_* from the total *κ*. The reduction in *κ_L_* with increasing Zn concentration can be ascribed to the enhanced phonon scattering owing to the mass field fluctuation and the disorder induced by the introduction of Zn at Mg sites. The undoped *κ_L_* follows the T^−1^ law very well, indicating the dominant anharmonic Umklapp processes [38], while the doped samples deviate from the T^−1^ law with a significant drop near room temperature. This is ascribed to the contribution from point defect scattering induced by the addition of Zn content and can be understood using the Callaway model [18,39]. At higher temperatures, the contribution from the point defect scattering becomes smaller compared to that from the Umklapp scattering. In addition, the existence of pores also contributes to the reduction in *κ_L_*. *κ_L_* contributes to most of the total *κ* in the lower temperature regime, while *κ_e_* increases with temperature and gradually becomes comparable to *κ_L_* in the higher temperature regime. Figure 5d shows the variation of *ZT* as a function of temperature. The undoped sample shows a peak *ZT* of 0.51 at 773 K, which is consistent with the results of Ref. [22]. The Zn-doped sample with *x* = 0.05 exhibits nearly identical *ZT* to the undoped sample, yielding a maximum *ZT* of 0.49. The doping of Zn for Mg within the polyanionic double-layers could not optimize the TE performance due to the reduced *PF* and the increased *κ* at higher temperatures in the Zn-doped samples. The values of *σ* increase and values of *κ_L_* decrease with increasing *x* in YbMg_1.85−*x*_Zn*_x_*Bi_1.98_, which is beneficial to *ZT*. However, Zn doping also significantly reduces *S* and causes an increase in *κ_e_* and a bipolar effect, which is detrimental to the *PF* and *κ*, consequently leading to a lower *ZT*.

## 4. Conclusions

A series of Zn-doped YbMg_1.85−*x*_Zn*_x_*Bi_1.98_ (*x* = 0, 0.05, 0.08, 0.13, 0.23) Zintl alloys have been prepared using high-frequency induction melting, ball milling, and SPS. XRD and SEM/EDS analysis of all the samples revealed that the matrix of the alloys belongs to the trigonal system with a $P\bar{3}m1$ (164) space group and a CaAl_2_Si_2_-type structure. The secondary phases are Yb_9_(Mg, Zn)_4.5−δ_Bi_9_ phases, and/or Bi and Bi-rich 1–3 phases. With increasing Zn concentration in *p*-type YbMg_1.85−*x*_Zn*_x_*Bi_1.98_, *σ* increases due to the electronegativity difference between Zn and Mg, and *κ_l_* decreases with increasing phonon scattering induced by more point defects together with the pores. However, the band gap decreases as more Zn is added, and bipolar conduction occurs, leading to a substantial decrease in *S* and therefore *PF*. The values of *κ* for samples with *x* ≥ 0.08 increase with temperature in the higher temperature regime, resulting in lower *ZT* values. The samples with *x* = 0 and 0.05 reach maximum *ZT* values of 0.51 and 0.49 at 773 K, respectively. It is suggested that the maximum amount of Zn doping is *x* ≤ 0.1, and synergistic optimization by co-doping at either site of the AMg_2_Bi_2_ Zintl compounds is essential for improving TE performance.

## Figures and Tables

**Table 1:** Refined structural parameters for the 1-2-2 phase in YbMg_1.85−*x*_Zn*_x_*Bi_1.98_ samples. The 1-2-2 phase has a space group $P\bar{3}m1$ (No. 164) and the following atomic Wyckoff positions: Yb, 1*a* (0, 0, 0); Mg/Zn, 2*d* (1/3, 2/3, *z*); and Bi, 2*d* (1/3, 2/3, *z*).

	*x* = 0	*x* = 0.05	*x* = 0.08	*x* = 0.13	*x* = 0.23
*a* (Å)	4.7341 (1)	4.7309 (2)	4.7264 (1)	4.7240 (1)	4.7145 (2)
*c* (Å)	7.6717 (2)	7.6717 (3)	7.6689 (3)	7.6675 (3)	7.6626 (3)
*V* (Å^3^)	148.901 (7)	148.70 (1)	148.360 (9)	148.187 (9)	147.492 (10)
*Occ. Mg/Zn*	0.925/0	0.875/0.025	0.885/0.04	0.845/0.065	0.815/0.115
*z* *_Mg/Zn_*	0.6268 (8)	0.6281 (11)	0.6261 (10)	0.6248 (10)	0.6247 (9)
*z_Bi_*	0.2441(2)	0.2461 (2)	0.2447 (2)	0.2458 (2)	0.2457 (2)
*ρ*_122_ (g/cm^3^) ^a^	7.208	6.983	6.985	7.032	6.959
*ρ_Bi_* (g/cm^3^) ^b^	9.814	9.824	9.802	9.827	9.819
*m*^122/Bi^ (wt. %)	85.3/14.7	89.3/10.7	83.6/16.4	82.6/17.4	88.3/11.7
*Exp. d* (g/cm^3^) ^c^	7.130	7.197	7.271	7.314	7.159
*% T. D.*	95.1	99.9	99.2	98.8	99.4
*Rp* (%)	8.91	12.1	10.8	11.4	11.6
*Rwp* (%)	11.9	17.1	15.4	15.9	17.2
*Rexp* (%)	3.79	3.20	3.41	3.65	3.30

^a,b^ Theoretical density for the 1-2-2 phase and the Bi phase obtained from the refinement of the XRD pattern, respectively. ^c^ Experimental bulk density obtained using Archimedes’ method.

## Data Availability

Data are contained within the article.
